# Differences in social interaction- vs. cocaine reward in mouse vs. rat

**DOI:** 10.3389/fnbeh.2014.00363

**Published:** 2014-10-17

**Authors:** Kai K. Kummer, Lena Hofhansel, Constanze M. Barwitz, Aurelia Schardl, Janine M. Prast, Ahmad Salti, Rana El Rawas, Gerald Zernig

**Affiliations:** ^1^Experimental Psychiatry Unit, Medical University of InnsbruckInnsbruck, Austria; ^2^Department of Psychology, University of InnsbruckInnsbruck, Austria

**Keywords:** dyadic social interaction, cocaine, conditioned place preference, Sprague Dawley rat, C57BL/6 mouse, alternative reward, addiction, substance use disorder

## Abstract

We previously developed rat experimental models based on the conditioned place preference (CPP) paradigm in which only four 15-min episodes of dyadic social interaction with a sex- and weight-matched male Sprague Dawley (SD) rat (1) reversed CPP from cocaine to social interaction despite continuing cocaine training, and (2) prevented the reacquisition/re-expression of cocaine CPP. In a concurrent conditioning schedule, pairing one compartment with social interaction and the other compartment with 15 mg/kg cocaine injections, rats spent the same amount of time in both compartments and the most rewarding sensory component of the composite stimulus social interaction was touch (taction). In the present study, we validated our experimental paradigm in C57BL/6 mice to investigate if our experimental paradigm may be useful for the considerable number of genetically modified mouse models. Only 71% of the tested mice developed place *preference* for social interaction, whereas 85% of the rats did. Accordingly, 29% of the mice developed conditioned place *aversion* (CPA) to social interaction, whereas this was true for only 15% of the rats. In support of the lesser likelihood of mice to develop a preference for social interaction, the average amount of time spent in direct contact was 17% for mice vs. 79% for rats. In animals that were concurrently conditioned for social interaction vs. cocaine, the relative reward strength for cocaine was 300-fold higher in mice than in rats. Considering that human addicts regularly prefer drugs of abuse to drug-free social interaction, the present findings suggest that our experimental paradigm of concurrent CPP for cocaine vs. social interaction is of even greater translational power if performed in C57BL/6 mice, the genetic background for most transgenic rodent models, than in rats.

## Introduction

In humans, substance dependence is accompanied by impaired social interactions. This negatively affects personal relationships and, if under treatment, the relationship with a psychotherapist, thus compromising treatment adherence (Grawe et al., 1994; Leichsenring et al., 2004). For the development of sorely needed novel therapeutic approaches, understanding the neurobiological mechanisms underlying drug- vs. social interaction reward is necessary.

We therefore developed an experimental model based on the conditioned place preference (CPP) paradigm in which only four 15-min episodes of dyadic (i.e., one-to-one) social interaction with a sex- and weight-matched male Sprague Dawley (SD) rat (1) reversed place preference from cocaine to social interaction despite continuing cocaine training, and (2) prevented the reacquisition/re-expression of cocaine CPP. In a concurrent conditioning schedule, pairing one compartment with social interaction and the other compartment with 15 mg/kg cocaine injections, rats spent the same amount of time in both conditioning compartments, suggesting that both stimuli possess the same reward strength (Fritz et al., 2011a,b). Further, we could show that the most rewarding sensory component of this composite stimulus social interaction was touch (Kummer et al., 2011).

In the last decades, considerable technical progress was made with the generation of genetically modified mice and their use in research, for example as reporter mice or for target-specific protein expression to activate or silence subpopulations of neurons using optochemical or optogenetic techniques (Ting and Feng, 2013). The application of such animal models is also highly desirable in the field of addiction research, and especially for the investigation of possible treatment strategies based on social interaction as an alternative reward to drugs of abuse (Zernig et al., 2013).

We therefore investigated if these transgenic mouse models could be used in our experimental paradigm, bearing in mind that compared to rats, male mice show a strict dominance-subordinance hierarchy together with enhanced conspecific aggression (Whishaw et al., 2001). These behavioral differences indicate that establishing dyadic social interaction as an alternative reward to drugs of abuse may be much more difficult in mice than in rats.

However, the present findings demonstrate that singly housed C57BL/6 mice can experience agonistic social interaction with a sex- and weight-matched male conspecific and develop place preference for the social interaction paired compartment, but to a lesser extent than SD rats. Still our experimental paradigms can employ the plethora of highly insightful transgenic mouse models with a C57BL/6 background to investigate the differential neurobiological basis of dyadic social interaction- vs. drug reward. Considering that human addicts regularly prefer drugs of abuse to drug-free social interaction, the present findings suggest that our experimental paradigm of concurrent CPP for cocaine vs. social interaction is of even greater translational power if performed in C57BL/6 mice, the genetic background for most transgenic rodent models, than in rats.

## Methods

### Animals

Male SD rats aged 6–8 weeks (weighing 150–200 g) were obtained from the Research Institute of Laboratory Animal Breeding of the Medical University Vienna (Himberg, Austria). Male C57BL/6N mice aged 6–8 weeks (weighing 20–22 g) were obtained from Charles River Laboratories (Sulzfeld, Germany). All animals were housed at a constant room temperature of 24°C and had *ad libitum* access to tap water and pelleted chow (Tagger, Austria). Experiments were performed during the light phase of a continuous 12-h light/dark cycle with the lights on from 0800 h to 2000 h. Animals were singly housed 7 days before the start of the behavioral experiments. The present experiments were approved by the Austrian National Animal Experiment Ethics Committee.

### Conditioned place preference apparatus

Conditioning of both SD rats and C57BL/6 mice was conducted in a custom-made three-chamber CPP apparatus (64 cm wide × 32 cm deep × 31 cm high) made of unplasticized polyvinyl chloride. The middle (neutral) compartment (10 × 30 × 30 cm) had white walls and a white floor. Two doorways led to the two conditioning compartments (25 × 30 × 30 cm each) with walls showing either vertical or horizontal black-and-white stripes of the same overall brightness and with stainless steel floors containing either 168 holes (diameter 0.5 cm) or 56 slits (4.2 × 0.2 cm each). Time spent in each compartment was digitally recorded with a video camera and analyzed offline with hand timers. The CPP apparatus was cleaned with a 70% camphorated ethanol solution after each session. All experiments were performed under neon ceiling light (58 W, 1 m distance) and radio-generated white noise.

For a set of mouse experiments the size of the conditioning chambers was halved by the insertion of opaque screens displaying the same visual cues as the respective conditioning chamber. The size of the neutral chamber was reduced to a third of the actual chamber size.

### Acquisition of cocaine CPP

For the acquisition of cocaine CPP, the conditioning procedure comprised a pretest session on day 1, eight consecutive training days in an alternate-day-design (one training session per day, a total of 4 training sessions each), and a CPP test on day 10. The stimuli were either (1) an i.p. injection of 15 mg/kg cocaine (pure base) for rats and mice respectively, or (2) only a saline injection. To emphasize, pretest-, training-, and CPP test sessions were of equal duration, i.e., 15 min. Pretest bias for any of the two conditioning chambers was declared if during pretest the animal spent more time in one of the conditioning chambers and the initially non-preferred chamber was subsequently paired with cocaine.

The dose of 15 mg/kg cocaine was chosen for both animals to ensure consistency between species, and represents a widely used dose for medium to high cocaine effects in both rats and mice (Witten et al., 2010; Kasahara et al., 2014).

### Acquisition of social interaction CPP

For the acquisition of social interaction CPP, the conditioning procedure comprised a pretest session on day 1, eight consecutive training days in an alternate-day-design (one training session per day, a total of 4 training sessions each), and a CPP test on day 10. The stimuli were either (1) dyadic social interaction, i.e., a 15-min dyadic social interaction session with a sex- and weight-matched male conspecific preceded by an i.p. injection of 1 or 10 ml/kg saline for rats and mice respectively, or (2) only a saline injection. To emphasize, pretest-, training-, and CPP test sessions were of equal duration, i.e., 15 min. Pretest bias for any of the two conditioning chambers was declared if during pretest the animal spent more time in one of the conditioning chambers and the initially non-preferred chamber was subsequently paired with social interaction. Animals that spent more time in the social interaction paired compartment than in the saline paired compartment during the CPP test were rated as showing conditioned place *preference* (CPP), whereas animals that spent less time in the social interaction paired compartment than in the saline paired compartment were rated as showing conditioned place *aversion* (CPA).

### Analysis of social interaction

To better understand which behavioral components of social interaction are most important for the demonstrated rewarding effect of social interaction in our paradigm, we performed a behavioral analysis of the recorded social interaction training sessions. For rats, we analyzed the recorded training sessions by measuring the time spent in direct contact, counting the number of nape attacks (“nape attacks” (Pellis et al., 1997) have also been termed “dorsal contact” (Panksepp et al., 1984) or “pouncing” (Trezza et al., 2010)) and pinning, episodes of genital sniffing, crawling under or over, allogrooming, boxing and biting. Nape attacks were defined as approaching and rubbing one’s snout into the interaction partner’s neck (Pellis et al., 1997). In adolescent rats, nape attacks are often followed by pinning, defined as a full rotation around the longitudinal axis of the animal’s body whose nape has been attacked, ending in a supine position with the other subject standing over it (see list of definitions in Trezza et al., 2010). Genital sniffing was declared if one rat sniffed the other rat’s anogenital area, whereas allogrooming involved grooming of all non-anogenital body areas. Crawling under or over the interaction partner, which can be interpreted as a form of “friendly” social interaction (Barnett, 1975), was also counted. Time spent in direct contact was defined as the time the two social interaction partners spent touching each other with any part of the body except the tail (social interaction partners almost always kept contact by intertwining or aligning their tails when not in contact with their rumps). In mice, we analyzed the recorded training sessions by measuring the time spent in direct contact, as well as for episodes of aggressive behaviors (i.e., attacks/fighting or biting).

### Acquisition of concurrent CPP for cocaine vs. social interaction

For the acquisition of concurrent CPP for cocaine vs. social interaction, the conditioning procedure comprised a pretest session on day 1, eight consecutive training days in an alternate-day-design (one training session per day, a total of 4 training sessions each), and a CPP test on day 10. The stimuli were either (1) cocaine, i.e., an i.p. injection of cocaine (cocaine doses of 15 / 5 / 1.7 / 0.17 / 0.05 mg/kg pure base, cocaine HCl diluted in saline), or (2) dyadic social interaction preceded by a saline injection. The initially non-preferred chamber was subsequently paired with social interaction. Animals that spent more time in the social interaction paired compartment during the CPP test were rated as showing CPP for social interaction, whereas animals that spent more time in the cocaine paired compartment were rated as showing CPP for cocaine. Following pharmacologic field convention, cocaine doses were diluted by a factor of three (i.e., close to half log_10_ steps) to cover a broad range of doses.

### Statistical analyses

Results are presented as group means ± standard errors (SEMs). Statistical analyses were performed with SPSS^1^ or GraphPad Prism^2^. For CPP experiments, data were analyzed using RM ANOVA followed by Holm-Sidak’s multiple comparisons test and effect sizes were calculated as Cohen’s d. Behavioral analysis was analyzed using Friedman test, Student’s *t*-test and Chi^2^ test where appropriate. The level of statistical significance was predefined at a *p* < 0.05, and the direction of the expected change was not set a priori, i.e., tests were always 2-sided.

## Results

### Social interaction CPP in SD rats

**Figure 1:**
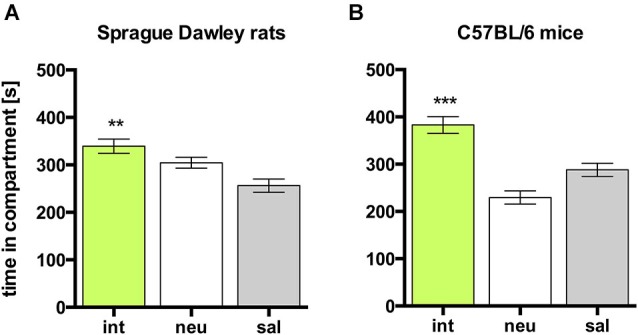
**Social interaction CPP in SD rats and C57BL/6 mice**. Times spent in the CPP apparatus compartments for **(A)** SD rats (*n* = 27) and **(B)** C57BL/6 mice (*n* = 42). Shown are group means ± SEM. Int, social interaction paired compartment; neu, neutral compartment; sal, saline paired compartment. **p < 0.01, ***p < 0.001 for time spent in int vs. coc (ANOVA, followed by *post hoc* test).

In rats, social interaction within the confines of the CPP apparatus produced an overall (i.e., group mean) place preference, as evidenced by a significant increase in time spent in the interaction paired (int) compartment, compared to the time spent in the saline paired (sal) compartment (Figure 1A; *n* = 27, ANOVA, *p* = 0.0043; int vs. sal, *p* = 0.0042, Cohen’s *d* = 1.11). When analyzing the individual animals, 23 of 27 rats (i.e., 85%) developed CPP for social interaction, whereas 4 rats (i.e., 15%) developed CPA for social interaction.

### Behavioral components of dyadic social interaction in SD rats

In rats, the recorded training sessions were analyzed for time spent in direct physical contact, the number of nape attacks and pinning, episodes of genital sniffing, crawling under or over, grooming, boxing and biting. From the first dyadic encounter on, the sex- and weight-matched rats spent more than 79 ± 0.8% (mean ± SEM) of the entire 15-min conditioning session in direct contact with each other. Time spent in direct contact remained at this high level across all four conditioning cycles (interaction session int1, 81%; int2, 80%, int3, 76%; int4, 78%; *p* = 0.30, Chi^2^ = 3.86). In addition, the total number of episodes for the different elements of social interaction did not change significantly across the conditioning sessions (see Table 1). The latter finding and the fact that there were hardly ever any episodes of hostile behavior, i.e., boxing or biting, suggests that the rats fully engaged in friendly (“agonistic”) social interaction from the first training session onwards and that the rats did not show habituation across the four conditioning cycles.

**Table 1:** **Behavioral analysis of social interaction in SD rats**.

Number of episodes	int1	int2	int3	int4	*p*	Chi^2^
**Nape attacks**	38 (6)	26 (3)	22 (4)	23 (2)	0.17	5.13
**Pinning**	4 (2)	4 (2)	3 (1)	4 (2)	0.96	0.35
**Genital sniffing**	9 (3)	6 (2)	11 (2)	6 (2)	0.094	6.31
**Crawling over/under**	20 (4)	22 (3)	24 (3)	26 (4)	0.74	1.41
**Allogrooming**	20 (3)	15 (4)	14 (4)	17 (1)	0.89	0.80
**Boxing**	1 (1)	1 (0)	2 (1)	2 (1)	0.75	1.67
**Biting**	0 (0)	0 (0)	0 (0)	0 (0)	n.a.	n.a.

*Mean number of episodes (±SEM) of different rat behaviors over the four social interaction conditioning sessions (int1–int4, n = 5 pairs, Friedman test statistics)*.

### Social interaction CPP in C57BL/6 mice

As in rats, social interaction conditioning in mice produced overall CPP, as evidenced by a significant increase in time spent in the interaction paired (int) compartment, compared to the time spent in the saline paired (sal) compartment (Figure 1B; *n* = 42, ANOVA, *p* < 0.0001; int vs. sal, *p* = 0.0020, Cohen’s *d* = 0.92). When analyzing the individual animals, 30 of 42 mice (i.e., 71%) developed CPP for social interaction, whereas 12 mice (i.e., 29%) developed CPA for social interaction.

When reducing the size of the conditioning chambers by a factor of two (i.e., from 750 to 375 cm^2^), mice also developed overall CPP (data not shown; *n* = 16, ANOVA, *p* = 0.0049; int vs. sal, *p* = 0.0024, Cohen’s *d* = 1.53). When analyzing the individual animals, 14 of 16 mice (i.e., 88% developed CPP for social interaction, whereas two mice (i.e., 12%) developed CPA for social interaction. Thus, the proportion of mice developing place preference was significantly higher in the mice conditioned in the smaller chambers (normal size, 71% vs. 29% in a total of 42 mice; reduced size 88% vs. 12% in a total of 16 mice; Chi square, *p* = 0.0029, Chi^2^ = 8.87). In contrast to CPP- vs. CPA development, the times spent in the social interaction paired compartment on CPP test day did not differ (unpaired *t*-test, *p* = 0.28).

### Behavioral components of dyadic social interaction in C57BL/6 mice

In mice, a subset of the recorded training sessions was analyzed for time spent in direct physical contact, as well as for number of attacks/fighting and biting (i.e., aggressive behaviors). Mice (*n* = 6) only spent 17 ± 3.6% of the entire 15-min conditioning session in direct contact with each other. Throughout all analyzed pairs, mice showed no signs of aggression, i.e., no attacks/fighting and no biting.

When reducing the size of the conditioning chambers by a factor of two, mice (*n* = 6) spent 22 ± 4.6% of the entire 15-min conditioning session in direct contact with each other. Also these animals showed no signs of aggression. Only in one case, animals engaged in “fierce ano-genital sniffing and grooming” which led to vocalizations of the badgered mouse. However this did not affect the formation of CPP for social interaction, as both mice spent more time in the social interaction paired compartment at CPP test.

Furthermore, mice in both conditions spent the same amount of time in direct contact with the social interaction partner (normal size, 17% of session time, reduced size, 22%; Chi square, *p* = 0.37).

### Comparison of social interaction CPP for SD rats and C57BL/6 mice

When analyzing the proportions of animals producing either place preference or place aversion, we found that significantly more rats than mice produced CPP for social interaction (rats, 85% / 15%, mice, 71% / 29%; Chi square, *p* = 0.017, Chi^2^ = 5.71).

Furthermore, rats spent significantly more time in direct contact with the social interaction partner (rats, 79% of session time, mice, 17%; Chi square, *p* < 0.0001, Chi^2^ = 77.00) than mice did.

### Comparison of cocaine CPP in SD rats and C57BL/6 mice

**Figure 2:**
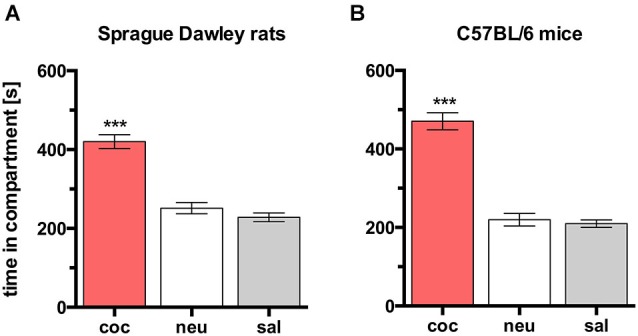
**Cocaine CPP in SD rats and C57BL/6 mice**. Times spent in the CPP apparatus compartments for **(A)** SD rats (*n* = 26) and **(B)** C57BL/6 mice (*n* = 8). Shown are group means ± SEM. Coc, cocaine paired compartment; neu, neutral compartment; sal, saline paired compartment. ***p < 0.001 for time spent in coc vs. sal (ANOVA, followed by *post hoc* test).

In both rats and mice, place preference conditioning with 15 mg/kg cocaine produced CPP for the cocaine paired compartment in all animals (Figure 2; rats, *n* = 26, ANOVA, *p* < 0.0001, coc vs. sal, *p* < 0.0001, Cohen’s *d* = 2.55; mice, *n* = 8, ANOVA *p* < 0.0001, coc vs. sal, *p* < 0.0001, Cohen’s *d* = 5.47).

### Concurrent CPP for social interaction vs. cocaine in SD rats

**Figure 3:**
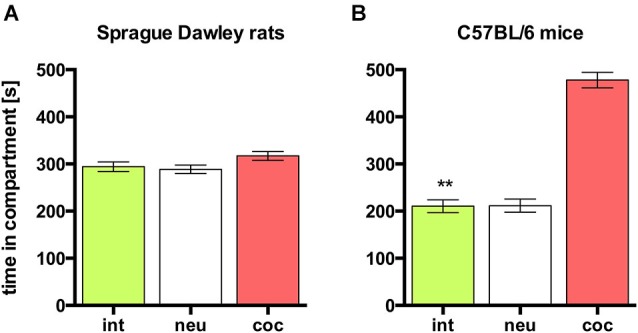
**Concurrent CPP for social interaction vs. 15 mg/kg cocaine in SD rats and C57BL/6 mice**. Times spent in the CPP apparatus compartments for **(A)** SD rats (redrawn from (Fritz et al., 2011b), *n* = 9) and **(B)** C57BL/6 mice (*n* = 16). Shown are group means ± SEM. Int, social interaction paired compartment; neu, neutral compartment; coc, cocaine paired compartment. **p < 0.01 for time spent in int vs. coc (ANOVA, followed by *post hoc* test).

In rats, concurrent place preference conditioning, i.e., pairing one compartment of the conditioning apparatus with dyadic social interaction and the other compartment with an i.p. injection of 15 mg/kg cocaine, produced no overall preference for either social interaction or cocaine. Animals spent the same amount of time in both conditioning compartments (Figure 3A; *n* = 9, ANOVA, *p* = 0.22; int vs. coc, *p* = 0.23). Thus, it seems that the reward strength (defined as the potency of a stimulus to produce place preference compared to other stimuli) of dyadic social interaction and 15 mg/kg cocaine is the same.

### Concurrent CPP for social interaction vs. cocaine in C57BL/6 mice: cocaine dose-response relationship

**Figure 4:**
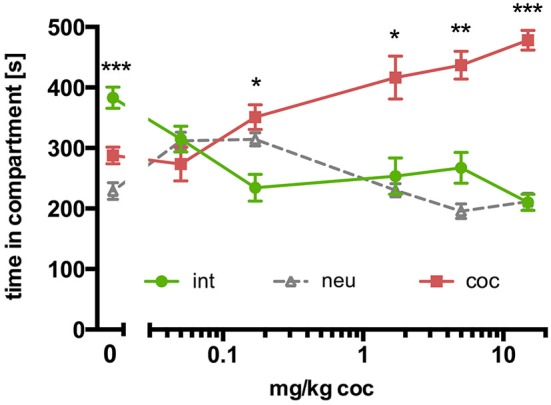
**Concurrent CPP for social interaction vs. cocaine in C57BL/6 mice: cocaine dose-response relationship**. Times spent in the CPP apparatus compartments for different doses of cocaine (15 mg/kg, *n* = 16; 5 mg/kg, *n* = 8; 1.7 mg/kg, *n* = 7; 0.17 mg/kg, *n* = 12; 0.05 mg/kg, *n* = 12; 0 mg/kg, *n* = 42). Shown are group means ± SEM. Int, social interaction paired compartment; neu, neutral compartment; coc, cocaine paired compartment. *p < 0.05, **p < 0.01, ***p < 0.001 for time spent in int vs. coc (ANOVA, followed by *post hoc* test).

In contrast to SD rats, concurrent place preference conditioning of C57BL/6 mice to dyadic social interaction and an i.p. injection of 15 mg/kg cocaine led to a pronounced preference for the cocaine paired compartment (Figure 3B; *n* = 16, ANOVA, *p* < 0.0001; int vs. coc, *p* < 0.0001, Cohen’s *d* = −4.44). Systematically reducing the training dose of i.p. cocaine from 15 to 0.05 mg/kg brought the time spent in the social interaction paired compartment closer and closer to the time spent in the cocaine associated compartment in an orderly fashion (Figure 4; 5 mg/kg: *n* = 8, ANOVA, *p* = 0.0004, int vs. coc, *p* = 0.0084, Cohen’s *d* = −2.48; 1.7 mg/kg: *n* = 7, ANOVA, *p* = 0.019, int vs. coc, *p* = 0.046, Cohen’s *d* = −1.87; 0.17 mg/kg: *n* = 12, ANOVA, *p* = 0.012, int vs. coc, *p* = 0.016, Cohen’s *d* = −1.59; 0.05 mg/kg: *n* = 12, ANOVA, *p* = 0.44, int vs. coc, *p* = 0.40, Cohen’s *d* = 0.48). Our findings indicate that for mice the reward strength of dyadic social interaction is comparable to the reward intensity of a 0.05 mg/kg i.p. cocaine injection.

To rule out the possibility that the size of the conditioning apparatus could have an effect on the reward strength of social interaction, we also performed concurrent place preference conditioning in chambers with reduced size. Also here mice developed place preference for the cocaine paired compartment (data not shown; *n* = 14, ANOVA, *p* < 0.0001; int vs. coc, *p* = 0.0003, Cohen’s *d* = −2.25).

## Discussion

In male young adult SD rats, dyadic social interaction with a sex- and weight-matched conspecific leads to the formation of CPP for social interaction in 85% of the conditioned animals, confirming previous findings obtained by previous generations of experimenters within our laboratory (Fritz et al., 2011b; Kummer et al., 2011; El Rawas et al., 2012) and by other independent groups (Peartree et al., 2012; Yates et al., 2013). Analysis of the behavioral components of this dyadic social interaction revealed that rats engaged in friendly (“agonistic”) social interaction with 79% of the conditioning session spent in direct physical contact. Further, the rats showed no signs of aggressive behaviors (i.e., boxing or biting). When applying the same conditioning schedule in an equally built conditioning apparatus to C57BL/6 mice, 71% of the animals developed a CPP for social interaction, whereas 29% developed CPA. Thus, for a 2-fold larger percentage of mice than rats, dyadic social interaction proved aversive. By reducing the size of the conditioning compartments this difference in the amount of animals producing CPA could be rescued. Analysis of the behavior revealed that mice only spent 17% of the conditioning sessions in direct contact, and interestingly this time did not change by reducing the size of the conditioning compartments. Further analysis revealed, that mice also did not show any kind of aggressive behavior (i.e., attacks or biting). However, despite the pronounced difference in time in direct physical contact, dyadic social interaction was, on average, a rewarding stimulus in both rodent genera.

We could recently show that when concurrently conditioning SD rats for social interaction (i.e., a natural reward/reinforcer) in one compartment and i.p. injections of 15 mg/kg cocaine (i.e., as a prototypical drug of abuse reward/reinforcer) in the other compartment, the animals spent the same amount of time in both conditioning compartments, suggesting that in SD rats both dyadic social interaction and 15 mg/kg cocaine have the same reward strength (Fritz et al., 2011b). Further, the rats’ CPP for cocaine vs. social interaction could be shifted seesaw-like by excitotoxic lesions of the nucleus accumbens shell (rendering cocaine more attractive), or the nucleus accumbens core and basolateral amygdala (rendering social interaction more attractive; Fritz et al., 2011a). When applying the same concurrent CPP schedule to C57BL/6 mice, we found that an i.p. injection of 15 mg/kg cocaine seems to have more reward strength than dyadic social interaction for this genus. By systematically lowering the cocaine dose, the time spent in the cocaine associated compartment approached the time spent in the social interaction associated compartment in an orderly fashion, until equilibrium was reached around 0.05 mg/kg cocaine. Therefore, in C57BL/6 mice social interaction seems to have the same reward strength as an i.p. injection of 0.05 mg/kg cocaine. The 300-fold (i.e., 15/0.05) rat/mouse difference in the relative reward strength of cocaine - when pitched against dyadic social interaction - is remarkable, but cannot be explained by pharmacokinetic differences, as both rats and mice show similar brain tissue concentrations of cocaine after intraperitoneal injections (Benuck et al., 1987; Pan et al., 1991). With respect to the translational power of our mouse experimental model for the human situation, it should be noted that rats are known among experimenters for persisting in their preference for natural rewards such as sweetness despite a history of extensive cocaine self-administration (Lenoir et al., 2007). Thus, an animal experimental model like the paradigm presented here, in which the individual, i.e., a mouse, finds cocaine (the drug of abuse) much more attractive than dyadic social interaction (the natural reward), is arguably of much higher translational power for the situation of the human addict who regularly prefers the drug of abuse over drug-free social interaction (Zernig et al., 2000, 2013) than a rat model, as our findings indicate that (1) a higher percentage of mice than rats find social interaction aversive and (2) cocaine has a roughly 300-fold higher relative (i.e., compared to social interaction) reward strength in mice than rats.

As a limitation, our paradigm only investigates one aspect of social interaction - albeit an important one - namely the CPP engendered by dyadic social interaction. In contrast to other researchers who focus on approach to a novel conspecific (e.g., Felix-Ortiz and Tye, 2014; Gunaydin et al., 2014) or hostile social interaction (e.g., Miczek et al., 2011), we concentrate on predominantly friendly (“agonistic”) social interaction with an increasingly familiar companion, thus modeling one of the most fundamental behaviors in human social cognitive development (Legerstee, 2009). In addition, by using a CPP-based paradigm, we achieve separation between the immediate neurobiological effects of social interaction (Gunaydin et al., 2014) or cocaine (i.e., its direct pharmacologic effects) and the conditioned (i.e., “psychological”) effects of social interaction vs. cocaine, thus providing increased differentiating power (Zernig et al., 2007) with our paradigm.

Finally, it should be pointed out that in this study we only investigated SD rats and C57BL/6 mice. Even though representing the two main mouse and rat strains used in animal experimentation (Johnson, 2012), it might be that other rodent strains behave differently in the experiments performed.

In conclusion, the present findings open the way for a transgenic mouse model-based neurobiological investigation of such strikingly different and therapeutically relevant stimuli that dyadic social interaction and cocaine present, for example, by employing either chemical-genetic approaches like designer receptors exclusively activated by designer drugs (i.e., DREADDs; Lee et al., 2014) or optogenetic approaches for activating and silencing the different neuronal subpopulations (Aston-Jones and Deisseroth, 2013). Thus, we can further investigate which neuron types of the nucleus accumbens (i.e., GABAergic projection neurons, also called medium spiny neurons or spiny projection neurons; cholinergic interneurons; different types of GABAergic interneurons; Kreitzer, 2009) are responsible for the therapeutically relevant switch in preference from the drug of abuse toward dyadic social interaction.

## Authors’ contributions

Gerald Zernig and Kai K. Kummer designed the experiments. Kai K. Kummer, Constanze M. Barwitz and Aurelia Schardl performed the experiments. Kai K. Kummer, Gerald Zernig and Lena Hofhansel analyzed the data. Kai K. Kummer and Gerald Zernig wrote the paper. Rana El Rawas, Ahmad Salti and Janine M. Prast have critically reviewed the contents of the paper and provided instrumental suggestions.

## Conflict of interest statement

The authors declare that the research was conducted in the absence of any commercial or financial relationships that could be construed as a potential conflict of interest.

## Footnotes

^1^www.ibm.com

^2^www.graphpad.com
